# Association of polycythemia vera with positive JAK2V617F mutation and myasthenia gravis: A report of two cases

**DOI:** 10.1002/ccr3.3574

**Published:** 2020-11-22

**Authors:** Sreethish Sasi, Mohamed A. Yassin, Sadat Kamran, Vazgen Mnatsakanyan

**Affiliations:** ^1^ Department of Internal Medicine Hamad General Hospital Hamad Medical Corporation Doha Qatar; ^2^ Department of Hematology National Centre for Cancer Care and Research Hamad Medical Corporation Doha Qatar; ^3^ Hamad General Hospital Neurosciences Institute Hamad Medical Corporation Doha Qatar

**Keywords:** interferon‐alpha, myasthenia gravis, paraneoplastic syndromes, polycythemia vera

## Abstract

Screening for MG in patients with PV positive for JAK2V617F mutation can help in early diagnosis and treatment, resulting in a significant reduction in morbidity and mortality.

## Graphical abstract

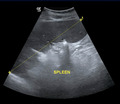

## 1 BACKGROUND

1.7% of myeloproliferative neoplasms are associated with autoimmune conditions. Association of myasthenia gravis (MG) with chronic myeloid leukemia is reported, but its association with polycythemia vera (PV) has never been reported. We report two patients who had MG and PV with JAK2V617F mutation. Both had splenomegaly but no thymoma.

Myasthenia gravis (MG) is an autoimmune disease characterized by antibodies to acetylcholine receptors at the neuromuscular junction (NMJ). The prevalence of MG in the United States is 0.02%.^1^ Prevalence in Arab countries is slightly higher (0.05%‐0.08%).^2^ It involves the extraocular muscles initially, characterized by fluctuating muscle weakness worsening with exercise and improving with rest. MG has an established association with autoimmune thyroiditis, Grave's disease, rheumatoid arthritis, systemic lupus erythematosus (SLE), and type 1 diabetes mellitus.^1^ Myeloproliferative Neoplasms (MPNs) are a group of rare blood cancers due to stem‐cell hyperplasia characterized by an increased peripheral blood cell count, overactive bone marrow, and proliferation of mature hematopoietic cells.^3^ Chronic myeloid leukemia (CML), essential thrombocythemia (ET), polycythemia vera (PV), and myelofibrosis are designated as MPNs, with CML being positive for BCR‐ABL1 gene fusion (Philadelphia chromosome)^4^ and the latter three negative.^5^ The majority of BCR ‐ABL1 negative MPNS are sporadic; however, there are reports of familial cases from different parts of the world.^6^ Paraneoplastic syndromes are clinical syndromes involving nonmetastatic systemic effects that accompany a malignant disease. Neurologic paraneoplastic syndromes are estimated to occur in fewer than 1% of patients with cancer.^7^, ^9^ There have been rare documentations of association of MG with CML.^8^, ^9^ However, the association of MG and PV has never been reported in the literature up to our knowledge.

## 2 CASE PRESENTATIONS

### 2.1 Case 1

**FIGURE 1:**
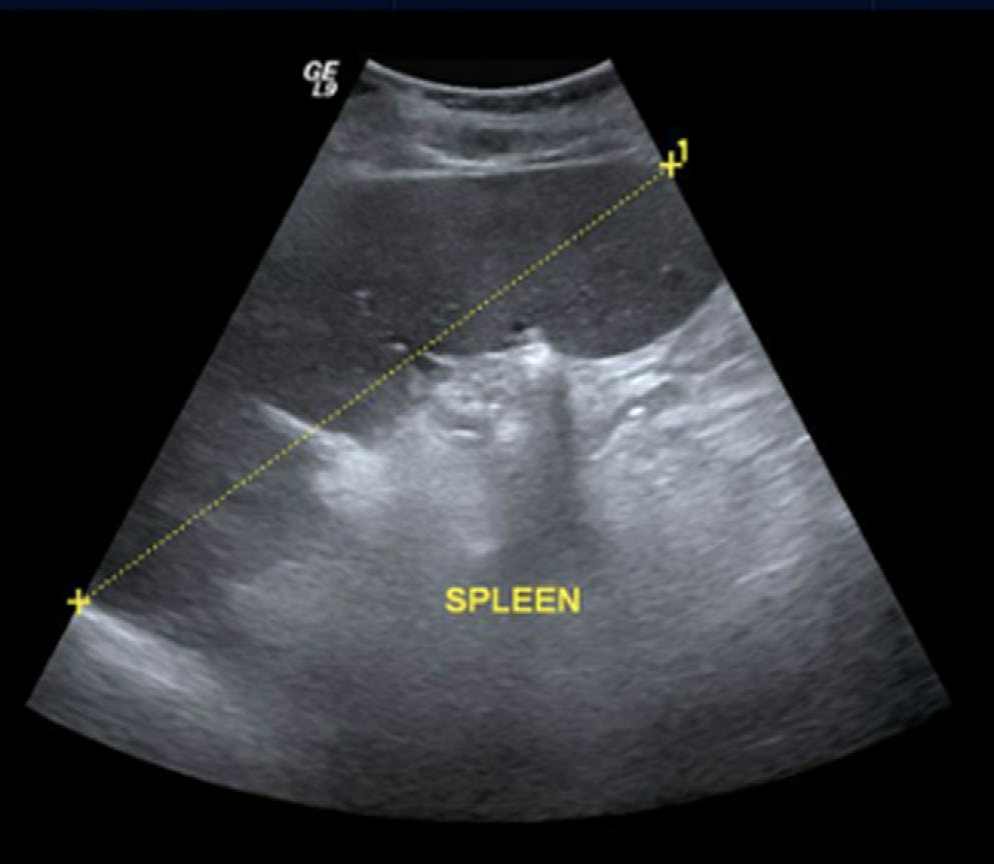
Ultrasound abdomen of Patient 1 showing massive splenomegaly

In March 2016, a 57‐year‐old lady presented with a 6‐month history of difficulty talking and voice change that started incidentally after an episode of shouting. She also complained of intermittent diplopia, which was more evident in looking toward the left side. There were no other associated symptoms. She did not report difficulty in swallowing, variation in the speech pattern, or difficulty in breathing. The past medical and family history was noncontributory. Her facial appearance, strained speech, and fatigue with recurrent effort suggested myasthenia gravis (MG). Electromyogram (EMG) along with positive high titer of antimuscle‐specific kinase (MuSK) antibodies (48.5 nmol/L, normal < 0.1 nmol/L) confirmed MG. Antiacetylcholine receptor (AchR) antibodies were negative. She was initially started on steroids and azathioprine to which she had a good response but developed steroid‐induced Cushing syndrome and multiple thoracolumbar spinal fractures. She was then shifted to tacrolimus with excellent response, and steroids were tapered. She developed congestive heart failure (CHF) with an ejection fraction of 25%, which was thought to be secondary to the tacrolimus. Tacrolimus was replaced by mycophenolate with bridge steroids for a short period. In the most recent clinic visit, she was doing well with mycophenolate. During her initial visit, she was incidentally noted to have high hemoglobin (17.8 mg/dL, normal < 16.5 mg/dL) with high hematocrit (64.3%, normal 35%‐45%), erythrocytes (6.9 × 10^6^/µL, normal 3.8‐4.8), leukocytes (17.4 × 10^3^/µL, normal 4‐10) and thrombocytes (505 × 10^3^/µL, normal 150‐400). A blood smear showed erythrocytosis with predominantly normochromic red cells, leukocytosis with neutrophilia, and thrombocytosis. Physical examination revealed hepatosplenomegaly, which was confirmed with an ultrasound abdomen showing a liver span of 19 cm and a spleen measuring 18 cm (Figure 1). She was diagnosed with Polycythemia Vera (PV) as per World Health Organization Diagnostic Criteria as JAK2V617F mutation was positive. Treatment was initiated with hydroxyurea 500 milligrams twice daily and aspirin 100 milligrams daily, with follow‐ups at regular intervals. The latest blood tests showed normal hemoglobin (14.8 mg/dL), erythrocytes (4.5 × 10^6^/µL), leukocytes (8.2 × 10^3^/µL), and thrombocytes (292 × 10^3^/µL) stable over the last 2 years.

### 2.2 Case 2

**FIGURE 2:**
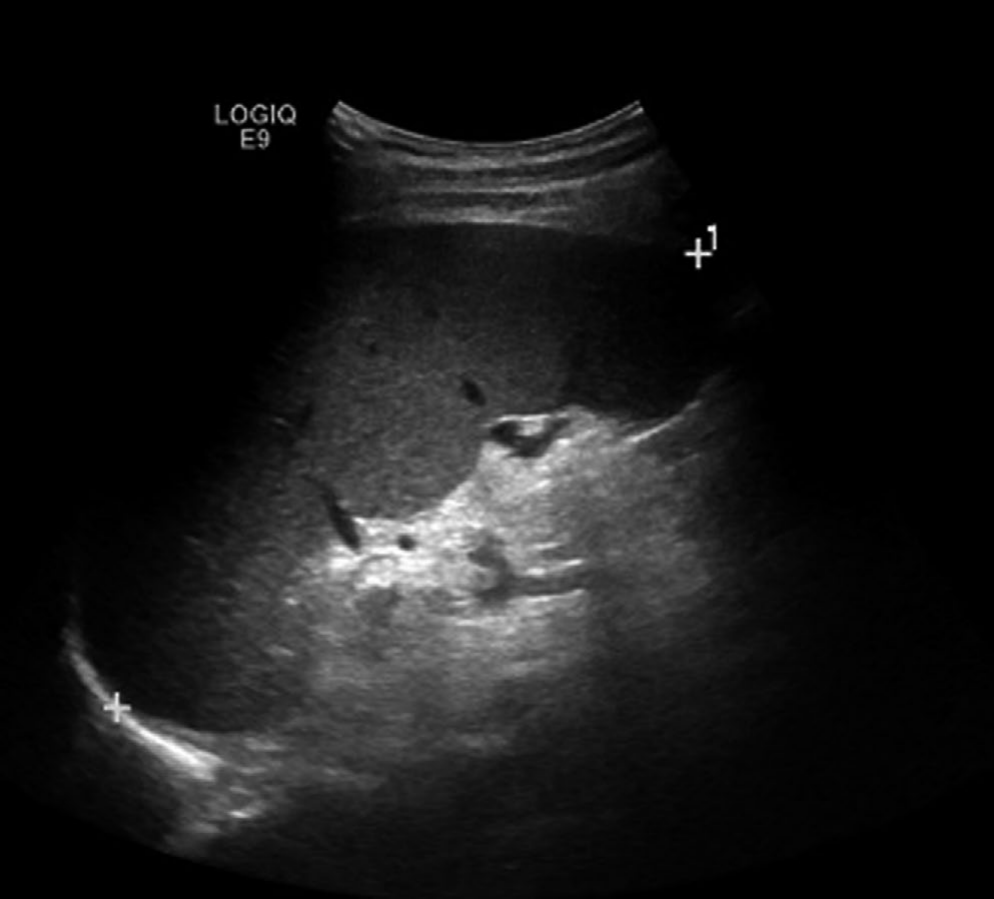
Ultrasound abdomen of Patient 2 showing massive splenomegaly

A 63‐year‐old gentleman, known to have hypertension was referred to hematology clinic in November 2018, after he was detected to have high hemoglobin (20.3 mg/dL, normal <16.5 mg/dL) with high hematocrit (62.6%, normal 35%‐45%), erythrocytes (7.2 × 10^6^/µL, normal 3.8‐4.8), leukocytes (14.3 × 10^3^/µL, normal 4‐10), and thrombocytes (674 × 10^3^/µL, normal 150‐400). A blood smear showed erythrocytosis with normal indices with a packed smear appearance, neutrophilic leukocytosis, and marked thrombocytosis. Ultrasound of the abdomen showed that the liver measured 14.4 cm, and the spleen measured 15.4 cm (Figure 2). He was positive for JAK2V617F mutation and was diagnosed with PV. Treatment was initiated with hydroxyurea 1 gram daily. Six months later, he was found to have pancytopenia during his routine follow‐up. He had a severely reduced hemoglobin of 2.9 mg/dL, a white cell count of 2.3 × 10^3^/µL, and a platelet count of 69 × 10^3^/µL. He also complained of diplopia when looking toward the left for a week. Peripheral smear showed no blasts, and repeated ultrasound showed a decrease in the size of the spleen. Hydroxyurea was stopped under the impression of drug‐induced bone marrow suppression; supportive transfusions were given, and blood counts were monitored. The neurologic evaluation showed that he had diplopia that becomes more pronounced on the left lateral gaze. There was a bilateral restriction of adduction and vertical movements of eyes. Pupils were equal and responsive to light. There was no facial asymmetry, and the gag reflex was preserved. Left‐sided ptosis was noted, which worsened with repeated movements of the eyelid. He developed hypophonia after counting out loud. The patient's son added that he has been having a deconjugate gaze and generalized weakness toward the end of the day for the last 2 years, but these symptoms were ignored. There was a high clinical suspicion of MG, and he was started on pyridostigmine 60 mg daily, observing for clinical response. There was an improvement in left eye ptosis over the next 3 days, and he was maintained on the same dose of pyridostigmine. AchR antibody was positive, but there was no thymoma on computed tomography (CT) of the thorax. Hydroxyurea was stopped, and he was maintained on close follow‐ups to monitor blood counts. Therapeutic venesection was done as and when needed. He was asymptomatic from the MG point of view and had normal blood counts during his latest clinic visit in June 2020.

## 3 DISCUSSION

Lymphoproliferative disorders are well known to be associated with autoimmune diseases (8% prevalence). MPNs are less commonly associated with autoimmune diseases (1.7% prevalence). U Dührsen et al described the spectrum of autoimmune diseases in 346 patients with MPNs, including 76 patients with CML, 46 with idiopathic myelofibrosis (IMF), 35 with PV, 42 with unclassifiable myeloproliferative disorders, 14 with myelodysplastic syndrome, and 133 with acute myelogenous leukemia (AML). They found no instances of MG preceding or during any of the MPNs. Autoimmune diseases such as rheumatoid arthritis, ankylosing spondylitis, and multiple sclerosis were associated with CML and pernicious anemia with IMF. They also described the spectrum of autoimmune diseases related to lymphoproliferative disorders, in which there was one case of MG associated with chronic lymphocytic leukemia (CLL).^7^

Paraneoplastic phenomenon in MPNs is rare. There are two case reports of patients who presented simultaneously with CML and MG.^8^, ^9^ Kumar et al in 2007 reported the case of a 47‐year‐old male who presented with diplopia and was found to have leukocytosis on routine laboratory evaluation. He was diagnosed simultaneously with CML and MG. He had splenomegaly (6 cm), BCR ‐ABL1 gene fusion, and positive anti‐AchR antibodies. He was started on steroids and pyridostigmine for MG and imatinib 400 mg daily for CML. Re‐evaluation after 12 weeks showed regression of spleen with a complete hematologic and cytogenetic response. There was a resolution of ptosis and ophthalmoplegia, and anti‐AchR turned negative.^8^ There are no cases of PV associated with MG reported so far. In our first case, both MG and PV presented simultaneously from the initial visit, whereas the second patient presented initially with PV, and MG manifested later, almost 6 months after initiation of treatment with hydroxyurea. There was no evidence of thymoma. Both patients were treated with hydroxyurea for their PV, but the second patient's course was complicated with pancytopenia. Patient 1 had positive anti‐MuSK antibodies, whereas patient 2 had positive anti‐AchR antibodies. There is no clearly defined pathophysiology in the literature regarding the association between PV and MG. The closest possible hypothesis is that of paraneoplastic syndrome. It is postulated that the anti‐AchR and anti‐MuSK autoantibodies specifically target the *α*3 subunit of nicotinic acetylcholine receptors (nAChRs), which are found in the thymus. It is proven that lung cancers and neuroblastomas can express the *α*3 subunit of nAChR and thus cause MG without thymoma.^10^ Patient 1 had a simultaneous presentation of both PV and MG. In patient 2, even though the MG was diagnosed 6 months after PV, there was a 2‐year history of diplopia, raising the possibility of simultaneous onset. Absence of thymoma, simultaneous onset, and positive anti‐AChR or anti‐MuSK antibodies are features suggesting possible paraneoplastic syndrome, in which PV expresses nAChRs.

Patients with PV who are more than 60 years of age and at high risk of thrombosis need cytoreductive therapy. The most common agent used for cytoreductive treatment in PV is hydroxyurea considering its cost‐efficacy and safety profile. But there is no evidence of the benefit of hydroxyurea in patients with MG. One of our patients developed MG after 6 months of therapy with hydroxyurea. There are no other reports in the literature showing the onset MG after hydroxyurea therapy. Other nontraditional agents that could be used for cytoreductive therapy are busulfan and interferon‐alpha. There are several case reports which link the onset of MG with busulfan therapy. Interferon‐alpha is an upcoming agent in cytoreductive therapy for PV.^11^ Some studies have shown that it is useful in the treatment of MG as well.^12^ A few case reports have shown the onset of MG after interferon‐alpha therapy.^13^, ^14^ It has been concluded that 6 months of IFN *α* therapy seems to be safe in MG, though in patients with malignancy, IFN *α* may cause increased autoimmunity, AChR positivity and MG.^12^

## 4 CONCLUSION

Any patient with a malignancy who develops a neuromuscular syndrome should be investigated for the possibility of paraneoplastic syndrome. Patients with PV positive for JAK2 mutation can develop MG as a paraneoplastic syndrome, in the absence of thymoma. PV shows good response to hydroxyurea therapy and MG to steroid plus cholinergic therapy. Interferon‐alpha is an upcoming modality for cytoreductive therapy in PV, which is also evidenced to bring about remission in MG.

## CONFLICT OF INTEREST

None declared.

## AUTHOR CONTRIBUTIONS

SS: Manuscript preparation, manuscript editing, and literature search. He will act as a study guarantor. MAY: Concept and idea, literature search, manuscript preparation, manuscript review and patient management. SK: Patient management, manuscript preparation and editing. VM: Patient management, manuscript review and editing.

## ETHICAL APPROVAL

Ethical approval was obtained from Medical Research Center at Hamad Medical Corporation (Approval Number: MRC‐04‐20‐867).

## Data Availability

Data sharing is not applicable to this article as no datasets were generated or analyzed during the current study.
